# Supplementary material for: The QTL and Candidate Genes Regulating the Early Tillering Vigor Traits of Late-Season Rice in Double-Cropping Systems
Source: Int J Mol Sci. 2024 Jan 25;25(3):1497. doi: 10.3390/ijms25031497 (PMC10855346; doi:10.3390/ijms25031497)
Supplement: Supplementary file 1 [file ijms-25-01497-s001.zip › ijms-2789224-supplementary.pdf]

Supplementary_Material

Supplementary Figures

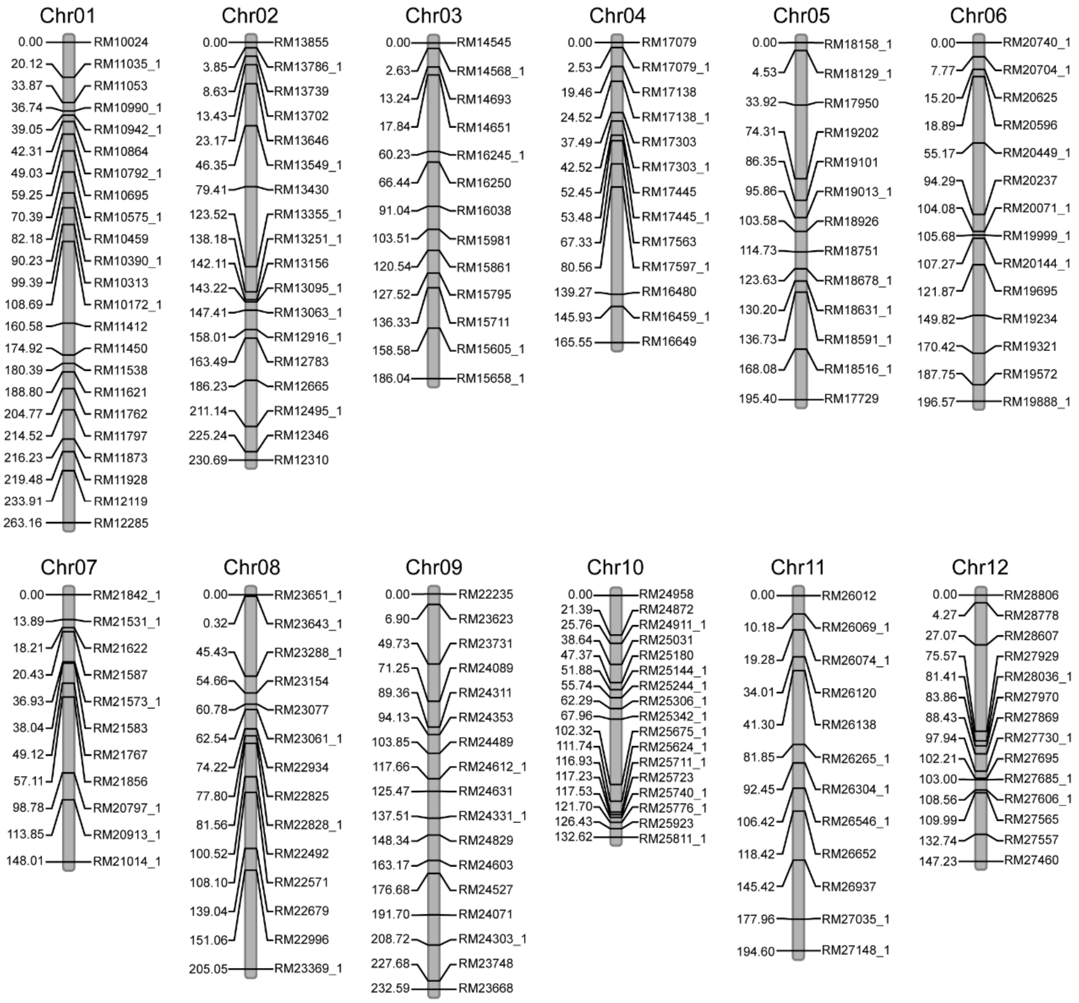

Figure S1. The genetic linkage map constructed by polymorphic SSR markers. SSR indicate simple sequence repeat.

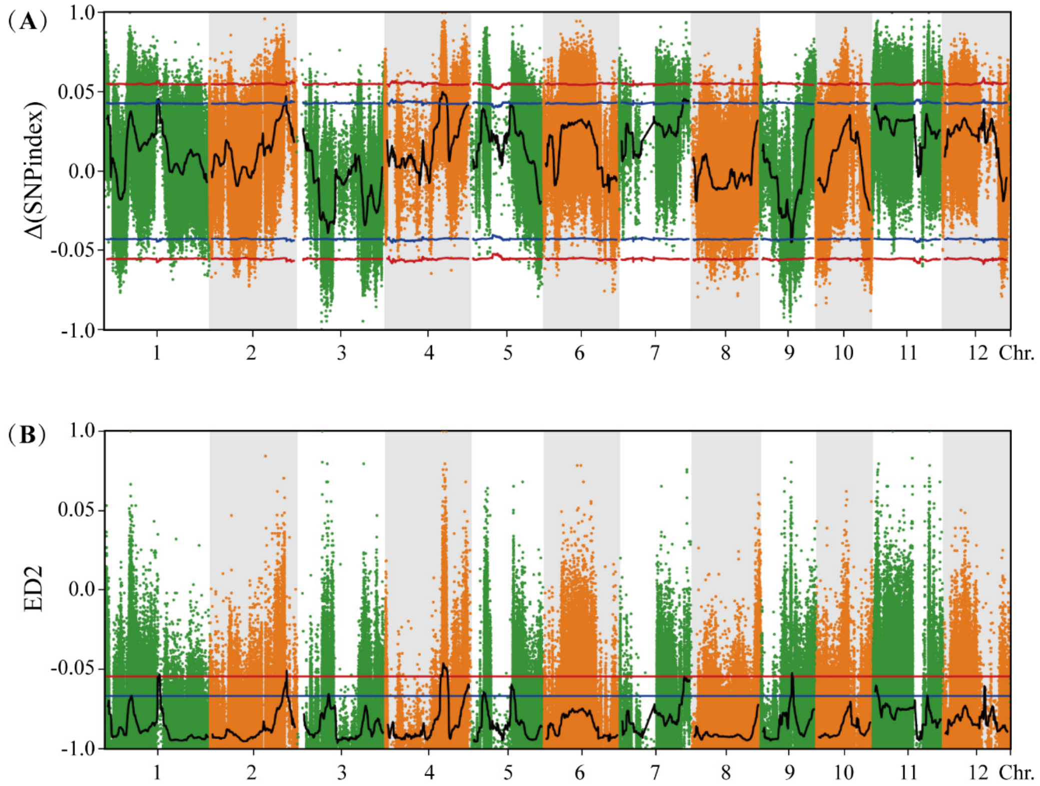

Figure S2. The QTLs determinate by SNP-index algorithm and ED algorithm. QTL indicates quantitative trait locus, SNP indicates single-nucleotide polymorphism, ED indicate Euclidean distance.

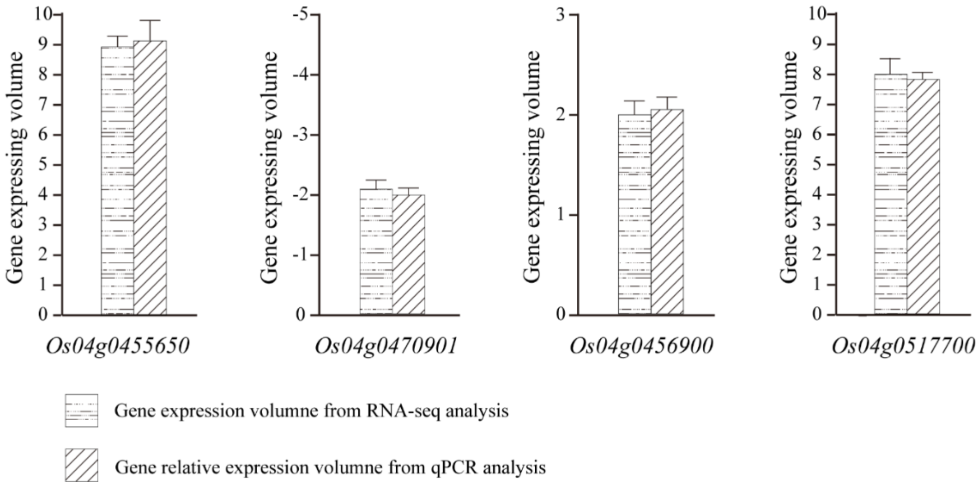

Figure S3. Genes expression pattern verified by qPCR. Four genes were randomly selected, and the gene expression pattern from RNA-seq analysis and qPCR analysis were shown in the same histogram. qPCR was performed according to our previous method (Liao et al., *BMC Genomics* 2015) and ACT1 (GenBank ID: AK100267) was the rice reference gene.

The relative expression level of each gene was calculated using the 2-(ΔΔCt) method as our previous method (Liao et al., *BMC Genomics* 2015). Three biological replicates were performed for the qPCR experiment.

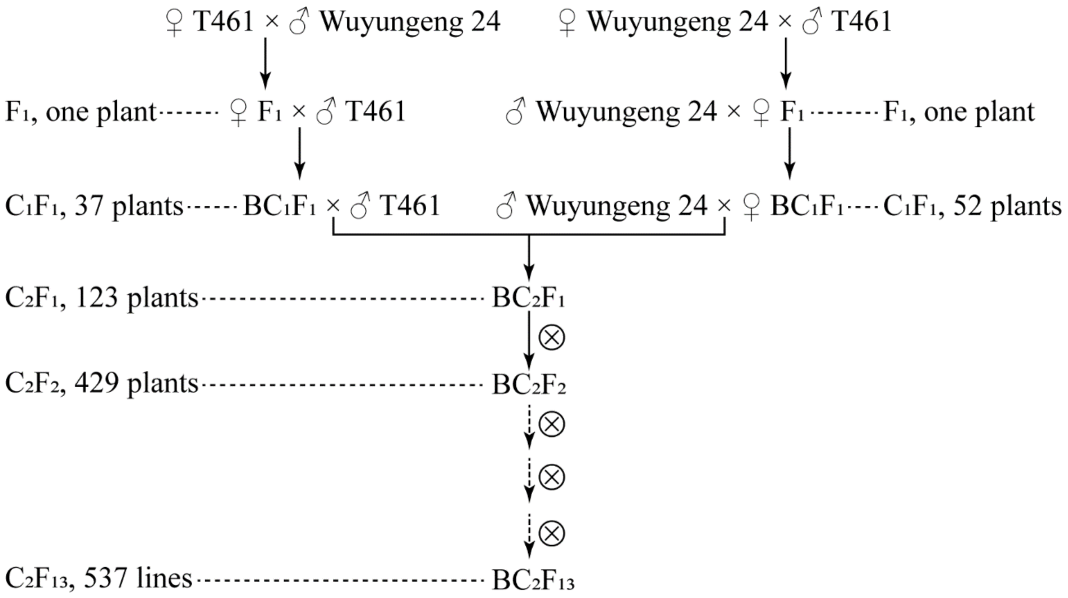

Figure S4. The flow chart of the BIL group construction. ♂ indicates male and ♀ indicates female; × and ⊗ indicate crossing and selfing, respectively.

Supplementary Tables

Table S1. Mapping results of RNA sequencing reads from rice tillering nodes.

Sample	RawData	CleanData	Clean data rate (%)	Total mapped reads (%)	Unique Mapped (%)	Multiple Mapped (%)	Unmapped (%)
SM1	45,942,422	43,797,536	99.57%	96.23%	92.56%	3.68%	3.77%
SM2	44,527,626	42,413,812	99.59%	96.17%	92.55%	3.62%	3.83%
SM3	48,058,002	45,437,930	99.56%	96.09%	92.43%	3.66%	3.91%
SM4	44,826,956	41,512,294	99.58%	95.79%	92.17%	3.62%	4.21%
TM1	53,053,264	50,990,864	99.58%	96.08%	92.42%	3.65%	3.92%
TM2	47,810,198	45,633,152	99.56%	95.96%	92.30%	3.66%	4.04%
TM3	48,308,170	45,955,010	99.59%	96.05%	92.40%	3.65%	3.95%
TM4	48,759,526	46,192,060	99.63%	96.07%	92.39%	3.68%	3.93%
Total	381,286,164	361,932,658	-	-	-	-	-

"SM" and "TM" indicate the mix sample of early-tillering rice lines and late-tillering rice lines; "1", "2", "3" and "4" indicate four biological replicates.

Table S2. Differentially expressed genes in the candidate region and their functional annotation.

Gene ID	Location (bp)	Symbol	Functional Description	$log_2FC(T/S)$
Os04g0455650	22795530-22796751	-	Conserved hypothetical protein	9.06
Os04g0470901	23527103-23527678	-	Hypothetical gene	-2.03
Os04g0456900	22834019-22835912	OsEIL6	Ethylene-insensitive 3, putative, expressed	2.04
Os04g0497300	24875271-24875935	-	ATOZI1, putative, expressed	9.74
Os04g0500600	24982051-24983036	-	Hypothetical protein	-4.21
Os04g0506800	25307143-25308493	-	Sialyltransferase family domain containing protein, expressed	2.61
Os04g0507400	25346878-25348347	-	Transferase family protein, putative, expressed	7.85
Os04g0517700	25884579-25885741	OsWAK51	OsWAK51 - OsWAK receptor-like protein kinase, expressed	7.98
Os04g0522500	26130711-26134356	OsGA2ox6	Gibberellin 2-beta-dioxygenase 7, putative, expressed	2.40
Os04g0534600	26720079-26721025	PEX11-4	Peroxisomal biogenesis factor 11, putative, expressed	2.44
